# Modeling and self-supporting printing simulation of fuse filament fabrication

**DOI:** 10.1038/s41598-024-67200-9

**Published:** 2024-07-15

**Authors:** Xiaohui Ao, Shengxiang Lin, Jianhua Liu, Huanxiong Xia, Junfeng Meng

**Affiliations:** 1 School of Mechanical Engineering, Beijing Institute of Technology, Beijing, 100081 China; 2 Tangshan Research Institute, Beijing Institute of Technology, Tangshan, 063015 China; 3 Hebei Key Laboratory of Intelligent Assembly and Detection Technology, Tangshan, 063015 China

**Keywords:** Additive manufacturing, Fused filament fabrication, Process modeling, Multiphase flow, Heat transfer, Mechanical engineering, Computational science

## Abstract

This study presented a comprehensive computational fluid dynamics-based model for fused filament fabrication (FFF) three-dimensional (3D) printing multiphase and multiphysics coupling. A model based on the framework of computational fluid dynamics was built, utilizing the front-tracking method for high precision of multiphase material interfaces, a fully resolved simulation at the mesoscale explores the underlying physical mechanism of the self-supported horizontal printing. The study investigated the influence of printing temperature and velocity on the FFF process, exhibiting a certain self-supporting forming ability over a specific range. The results indicated that during the printing of large-span horizontal extension structures, the bridge deck material transitions from initial straight extension to sagging deformation, ultimately adopting a curved shape. The straight extension distance is inversely proportional to the depth of the sagging deformation. Additionally, the study revealed that printing temperature primarily affected the curing time of the molten material, while printing velocity fundamentally affected the relaxation time of both thermal and dynamic characteristics of the material.

## Introduction

Fused filament fabrication (FFF) has been a prominent additive manufacturing technology since its proposal in 1988. Despite extensive research through experiments^1,2^, theoretical analyses^3,4^, numerical simulations^5–7^, and other means^8–10^, elucidating the underlying mechanisms of FFF remains a challenge. The high cost and limited generality of experimental approaches necessitate a deeper exploration through theoretical modeling and numerical simulations. While previous research has focused on partial physical effects, there is a scarcity of comprehensive modeling and integrated simulations addressing multiphase and multiphysics coupling.

Furthermore, due to the complexity of the process, theoretical modeling and numerical simulations are relatively limited compared with experiments. Bellini^11^, for instance, modeled the flow and heat transfer of ceramic material during extrusion and deposition. Despite the two-dimensional (2D) of the established model, it proves to be relatively comprehensive. Crockett^12^ and Middleman^13^ treated the deposition and spreading process of molten material as laminar axisymmetric flow, utilizing the Bingham viscosity model for simulation. Furthermore, while an ideal boundary of complete wetting was assumed, their work holds significant reference essential for studying the spreading behavior of melts in FFF. Park et al.^14^ and Duineveld^15^ explored the spreading process utilizing the Frenkel model based on energy balance. Their results correlated with the momentum conservation-based modeling method. The fusion of adjacent materials upon deposition was influenced by temperature, a crucial factor in determining the strength of the fuse. Thomas and Rodriguez^16^ proposed a simplified 2D heat transfer model based on the rectangular cross-section of the deposited filament. They derived the temperature distribution equation, indicating that the temperature gradient along the cross-section dissipated shortly after deposition. Subsequently, based on this finding, Bellehumer et al.^17^ assumed a uniform temperature distribution on the cross-section, resulting in a more simplified one-dimensional model.

The modeling of process and material behavior above provided a mathematical basis for simulation implementation. Many researchers^18–20^ use the finite element method combined with birth–death element to simplify simulating temperature and stress fields in the FFF process. Furthermore, while the precision of simplified finite element analysis may not be high, comparing simulation results from multiple schemes can aid in designing optimized part structures and the selection of process parameters. Domingo-Espin et al.^21^ established a finite element simulation framework using ANSYS software for numerical research on the FFF printing process. The result underscored the nozzle movement path (scanning path) during printing affecting the quality of the final part. Zhou et al.^22^ studied the evolution process of deposition morphology and temperature distribution of filament material based on the continuum theory, indicating that the thermal conductivity and specific heat capacity of the filament were nonlinear with temperature and phase state. Literature analysis indicates that the current modeling and simulation efforts in FFF 3D printing focused on partial physical effects and phenomena in local space or time, with limited research on comprehensive modeling and integrated simulation for multiphase and multiphysics coupling^23,24^.

Self-supporting printing of overhang structures is a prominent area of FFF research. To ensure the successful printing of the overhang structure, it is a common practice to add auxiliary support structures where necessary^25–29^. However, the printing and removal of the auxiliary support structure not only affect the surface quality of the part but also reduce the efficiency of forming, resulting in additional material consumption. For instance, Strano et al.^30^ employed a purely mathematical 3D implicit function to design and generate support structures with hierarchical features, utilizing the forming direction of the part and the honeycomb-shaped support structure as an entry point for their research. The graded honeycomb support structure was designed to solid support in areas where weight concentration occurs while minimizing support usage in other regions. Furthermore, due to the simple representation of the geometric structure of the part using pure mathematical expressions, this method exhibited exceptional universality. Paul and Anand^31^ analyzed part construction directions’ influence on cylindricity and flatness errors, developing a voxel-based approach to minimize shape errors and maximize material consumption. Subsequently, Vaidya and Anand^32^ proposed an optimal support structure generation method combining cellular structures with Dijkstra’s shortest path algorithm, aiming to minimize support material use while considering internal support accessibility^33–36^.

This study models the FFF process, conducting a simulation investigation of the self-supporting printing of overhang structures. In section "Numerical method", the thermal and mechanical properties of the material, the phase interface, and other important physical phenomena during printing were modeled. Section "Problem formulation" proposed the concept of overhang feature structure and analyzed horizontal extension self-supporting printing characteristics. Section "Results and discussions" implemented simulations on horizontal extension printing, exploring the influence of printing temperature and velocity on internal physical fields and material behaviors. Finally, Section "Conclusion" provided a summary of the primary conclusions of the study.

## Numerical method

The working principle of FFF is easy to understand. However, the mechanism of multiphase and multiphysics coupling is quite complex. Similar to other flow and heat transfer processes, the behavior of ambient gas, molten material, and solid material during printing is governed by three fundamental laws: mass, momentum, and energy conservation. Because the surrounding pressure of the ambient gas and the material extruded from the nozzle remains approximately constant, and the fluid velocity is very low, the flow was considered incompressible. Additionally, the shear rate in the forming domain was relatively small, and thus the viscous heating term was ignored. The governing equations were written as:

1 $$ \frac{\partial \rho }{{\partial t}} + \nabla \cdot \left( {\rho {\mathbf{u}}} \right) = S_{q} $$

2 $$ \frac{{\partial \left( {\rho {\mathbf{u}}} \right)}}{\partial t} + \nabla \cdot \left( {\rho {\mathbf{uu}}} \right) = - \nabla p + \nabla \cdot \mu \left( {\nabla {\mathbf{u}} + \nabla {\mathbf{u}}^{T} } \right) + \rho {\mathbf{g}} + {\mathbf{S}}_{m} $$

3 $$ c_{p} \frac{{\partial \left( {\rho T} \right)}}{\partial t} + c_{p} \nabla \cdot \left( {\rho {\mathbf{u}}T} \right) = \nabla \cdot \left( {\lambda \nabla T} \right) + S_{e} $$

Here, *t* is the time;* ρ*, *µ*, *λ*, and *c*_p_ are the density, viscosity, thermal conductivity, and specific heat capacity, respectively; **u** represents the velocity vector, *p* is the pressures, **g** is the gravity acceleration, and *T* denotes the temperature field. *S*_*q*_, **S**_*m*_, and *S*_*e*_ denote the mass source term, momentum source term, and internal heat source term.

As a typical multiphase flow process, accurately tracking the phase interface motion is key to modeling FFF multiphase flow. To accurately describe the behavior of the phase interface during the process, the finite volume/front-tracking method^37,38^ was used to model the multiphase flow. The phase interface is represented by connected marker points, typically referred to as the “front”, which is advanced by interpolating the velocity from the fixed grid. The front serves the purpose of constructing a marker function, denoted as *I*, identifying the different phases. Subsequently, the material property at each grid point is determined using the marker function:

4 $$ \varphi = \varphi_{a} I{ + }\varphi_{p} \left( {1 - I} \right) $$

where *φ* denotes a generalized material property. Marker function *I* is 0 inside phase *p*, and 1 inside phase *a*, with a smooth transition across the interface.

The finite volume/front-tracking method discretizes the computational domain using Euler grids and constructs the interface using Lagrangian points. For the momentum equations, the source **S**_*m*_ resulting from the surface tension force of the molten material needs to communicate the information from the Lagrangian points to the Euler grids as:

5 $$ {\mathbf{S}}_{m} = \sigma \int_{F} {k_{f} {\mathbf{n}}_{f} \delta \left( {{\mathbf{x}} - {\mathbf{x}}_{f} } \right)} {\text{d}}A_{f} $$

Here, *σ* is the surface tension coefficient, *k*_*f*_ denotes twice the mean curvature in three dimensions, **n**_*f*_ is a unit vector normal to the material surface pointing into the material. **x** and **x**_*f*_ are the coordinates of the Euler grid and the Lagrangian points, respectively. *δ* is a three-dimensional (3D) delta function, generally the product of three one-dimensional functions:

6 $$ \delta \left( {{\mathbf{x}} - {\mathbf{x}}_{f} } \right) = d\left( {x - x_{f} } \right)d\left( {y - y_{f} } \right)d\left( {z - z_{f} } \right) $$

Here, the one-dimensional function *d* is given as a Peskin cosine interpolation function:

7 $$ d\left( r \right) = \left\{ {\begin{array}{*{20}c} {\left( {{1 \mathord{\left/ {\vphantom {1 4}} \right. \kern-0pt} 4}h} \right)\left( {1 + \cos \left( {{{\pi r} \mathord{\left/ {\vphantom {{\pi r} 2}} \right. \kern-0pt} 2}h} \right)} \right)} & {\left| r \right| \le 2h} \\ 0 & {\left| r \right| > 2h} \\ \end{array} } \right. $$

where *h* denotes the size of the local Euler grids. To consider the ambient gas flow due to temperature difference, the Boussinesq approximation was used to model natural convection^23^.

The nozzle is an important component in the FFF 3D printer, which melts and extrudes the forming material. The nozzle structure of different printers varies, and it primarily influences the extrusion of melted material. In contrast, the deposition and spreading following extrusion are less constrained by it. This study focused on the deposition and spreading characteristics of the molten material after flowing out of the nozzle, using a volume source with a specific mass and heat intensity to stimulate the function of the nozzle. Specifically, the volume source is represented by a moving Lagrangian point with a certain volume as:

8 $$ S_{q} = \rho \dot{\Phi }\delta \left( {{\mathbf{x}} - {\mathbf{x}}_{s} } \right) $$

9 $$ S_{e} = \rho c_{p} T_{inj} \dot{\Phi }\delta \left( {{\mathbf{x}} - {\mathbf{x}}_{s} } \right) $$

**Figure 1:**
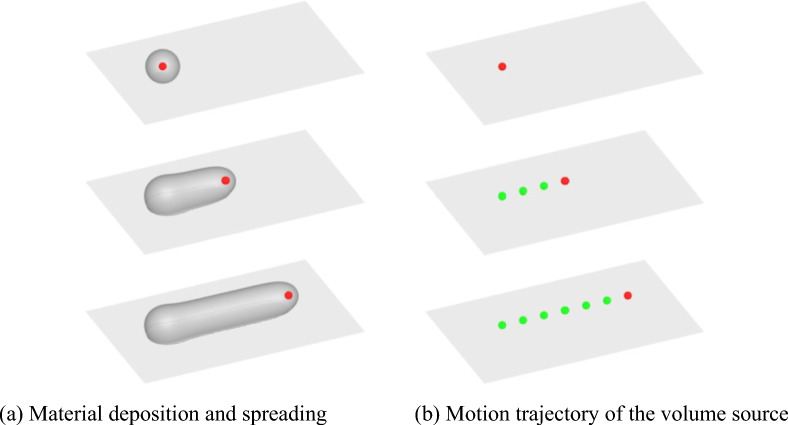
The equivalent nozzle model.

Here, $\dot{\Phi }$ and *T*_*inj*_ represent the volume rate and temperature of the moving Lagrangian point, respectively, and **x**_*s*_ denotes its coordinate position. As shown in Fig. 1, the red volume source moved along a specified path and continuously “extruded” high-temperature molten polymer material. Given the motion velocity *U*_*s*_ and injection rate *Q* for the nozzle, the nominal diameter $D=\sqrt{4Q/\pi {U}_{s}}$ of the deposited material. Hence, the volume rate $\dot{\Phi }=Q/\frac{4}{3}\pi {R}^{3}$, where *R* is the radius of the volume source, given as 0.2*D* in the simulations.

**Figure 2:**
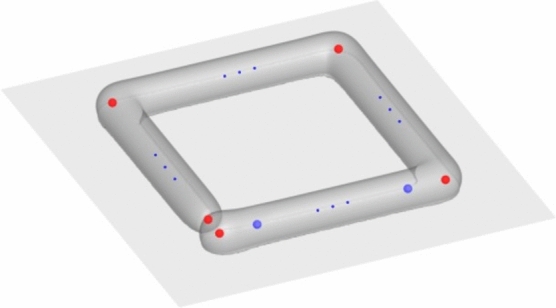
Volume source trajectory settings.

The setting of the motion trajectory of the volume source is a crucial part of the model, determining the deposition path of the molten material. As shown in Fig. 2, when the material was deposited along a straight line, its starting and ending points were two crucial trajectory points. If the material was deposited along a curve, the corner of the curve was another crucial trajectory point. Other trajectory points (blue dots) between these crucial points (red dots) were optional auxiliary points. Notably, in the case of continuous bending, the more trajectory points are arranged, the higher the geometric accuracy is obtained.

**Figure 3:**
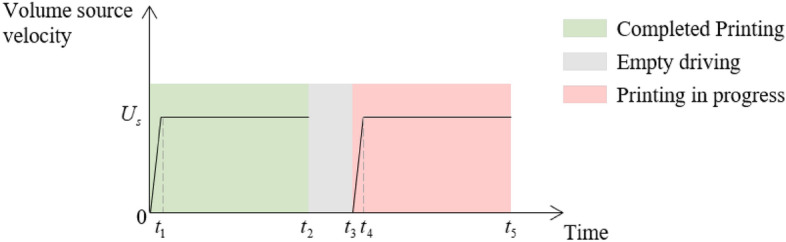
The working state of the volume source.

Figure 3 schematically depicts the working state of the volume source during manufacturing. Upon the start of each printing, the volume source went through a stage of uniform acceleration until it reached the preset velocity (0–*t*_1_ and *t*_3_–*t*_4_). This acceleration process not only brought the simulation closer to reality but also benefited the stability of numerical solutions. After completing the printing of one path, the volume source drove the toolhead to the starting point of the next path (*t*_2_–*t*_3_). In the equivalent nozzle model, such an empty driving process did not occur, and the volume source appeared directly at the designated location. However, such a time interval was necessary to facilitate the heat transfer and morphology development of the deposited material. In the simulation, the time step size corresponded to the moving speed of the volume source, ensuring that the volume source movement distance within every time step does not exceed the space grid size.

The phase states of the material vary at different temperatures, exhibiting different material behaviors. This study was based on the primary common physical behavior of various polymer materials during printing, considering cooling/solidification, volume change, and solid stress. For a detailed modeling process of the material behavior, please refer to our published papers.

## Problem formulation

**Figure 4:**
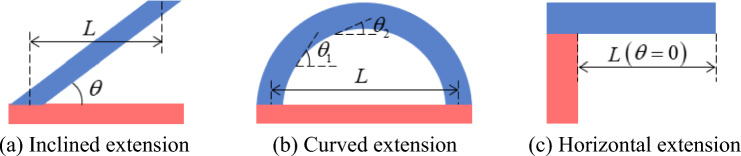
Overhang feature structures.

The FFF process melted the fed solid thermoplastic polymer via heating while depositing and spreading them along a specified path. The layer-by-layer fabrication nature required that the current printing layer was supported using a substrate or previous layer to prevent the collapse of the melted material under vertical downward forces (such as gravity) during manufacturing. The three types of structures leading to fabrication failure in Fig. 4 were collectively referred to as overhang feature structures. These were defined as long-span areas hanging over the part void, where there was a self-supporting structure below.

From Fig. 4a–c the difficulty of self-supporting fabrication of each overhang feature structure varied, with the angle of inclination θ and the length of extension L of the overhang feature structure being two key structural parameters. Here, the inclination angle θ is the angle between the tangent of the structure surface and the horizontal line. The force analysis indicated that the smaller the inclination angle *θ* or the longer the extension length *L*, the more difficult it was for the molten material to get effective self-support during manufacturing. Additionally, the inclination angle was more crucial than the extension length for the process. The inclination angles of different areas of the overhang feature structure in Fig. 4b were different, while the inclination angles of Fig. 4a and c were fixed. In Fig. 4c, with an inclination angle of 0 formed a horizontal extension, and it was the most challenging type of overhang feature structure.

**Figure 5:**
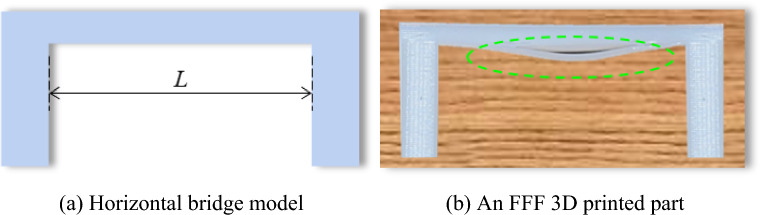
A schematic of a horizontal extension structure.

This study aimed to explore the self-supporting ultimate forming ability of the FFF process for horizontal extension structures. Notably, the horizontal bridge model in Fig. 5a was designed as the research object. The deflection of different positions on the bridge deck was different, and its size correlated with the span of the bridge deck (i.e., the length *L* of the bridge deck), the size of the cross-section, and the physical property of the material. If the horizontal bridge model was simplified as a simply supported beam, the deflection reached its maximum at the midpoint:

10 $$ W_{\max } = \frac{{5qL^{4} }}{384EI} $$

Here, *q* denotes the uniformly distributed load on the beam and *EI* is the bending stiffness of the beam (*E* and *I* represent the elastic modulus and cross-sectional moment of inertia, respectively). From Eq. (6), it was observed that the longer the span *L*, the larger the deflection near the midpoint of the bridge deck. Under large deflection, the tendency of the molten material to bend during the printing process became stronger. As shown in Fig. 5b, when printing from bottom to top, the material at the bottom of the bridge deck experienced sagging under the action of gravity.

The polymer used in the FFF process presents a strong viscous behavior even in the molten state, which is beneficial to self-support formation. However, compared with the solid state with a regular molecular arrangement, the intermolecular force inside the molten material is relatively weak, which allows it to withstand a certain external force. As the molten material is generated, its temperature is higher than the glass transition point, and it is in a viscous-fluid state with a very low elastic modulus. The intermolecular forces inside the viscous-fluid material are weaker than those in its solid state. At this point, if the deflection of the deposited material is extremely large, its internal forces cannot balance the gravity force, thus bending the molten material. As shown in Fig. 5b, the forming state of the bottom material of the horizontal bridge deck was the most critical, supporting subsequent materials, and determining the overall quality of the part.

**Figure 6:**
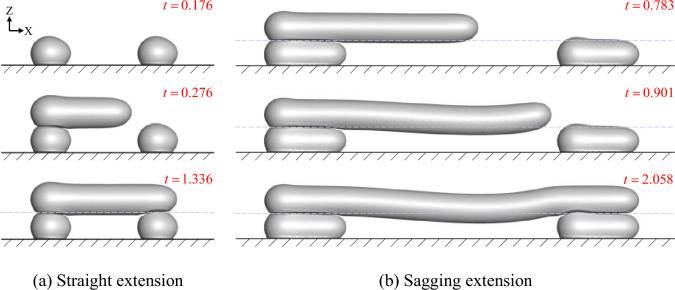
Horizontal bridge self-supporting fabrication.

Figure 6 shows the deposition and spreading of the first filament on the bridge deck with different spans, where the horizontal light blue dashed line is the reference line. Figure 6a depicts that the molten material exhibited a specific self-supporting forming ability over a specific span. After the deposition was completed and cooled for 1 s, there was insignificant deformation of the material, spanning horizontally without external support at the bottom. Increasing the span, the molten material in Fig. 6b underwent vertical deformation after stable extension to a certain distance. At the end of the deposition and cooling for 1 s (*t* = 2.058), the horizontally spanning material on the deck exhibited some form of deflection. This correlated with the sagging pattern in Fig. 5b.

The deposition and spreading of the first filament on the bridge deck were not affected by other materials (except for the piers), and the formation of its spreading morphology was a “free” development process, and self-supporting forming was the most difficult. Studying the ultimate forming ability of horizontal extension of molten materials (i.e., the maximum length of straight extension) provided a theoretical basis for the design of auxiliary support structures during printing. Upon optimization, the spacing of external support structures was essential not only for reducing material usage but also for ensuring the efficiency of fabrication.

## Results and discussions

In the simulations, the size of the computational domain is 15*D* × 4*D* × 4*D* with a grid resolution of 450 × 120 × 120, thus fixing the numerical resolution at *D*/30; the nominal diameter of the deposited filament is maintained at 0.25 mm, and thus the injection volume rate *Q* must be varied at different nozzle velocities *U*_s_ according to the equation $D=\sqrt{4Q/\pi {U}_{s}}$. The flow and heat transfer boundary conditions are listed in Table 1. In a three-dimensional computational domain, the bottom is a plate with a thickness of 5 mm and a controlled temperature of 40 °C. The four sides and the top of the domain are open, and the ambient gases can freely enter and exit as the process proceeds. The initial velocity of the gas inside the domain is zero. The ambient temperature *T*_0_ is given as 20 °C and its convection heat transfer coefficient *h* is 20 W/(m^2^K). These simulations were run on the homemade FORTRAN code.

**Table 1:** The flow and heat transfer boundary conditions.

Position	Flow	Heat transfer
Bottom	**u** = 0	$\partial T/\partial n = 0 \& \;T = 40 {}^{{\text{o}}}{\text{C}}$
Sides	$\partial {\mathbf{u}}/\partial n = 0$	$- \lambda \left( {\partial T/\partial n} \right) = h\left( {T - T_{0} } \right)$
Top	*p* = constant	$- \lambda \left( {\partial T/\partial n} \right) = h\left( {T - T_{0} } \right)$

**Figure 7:**
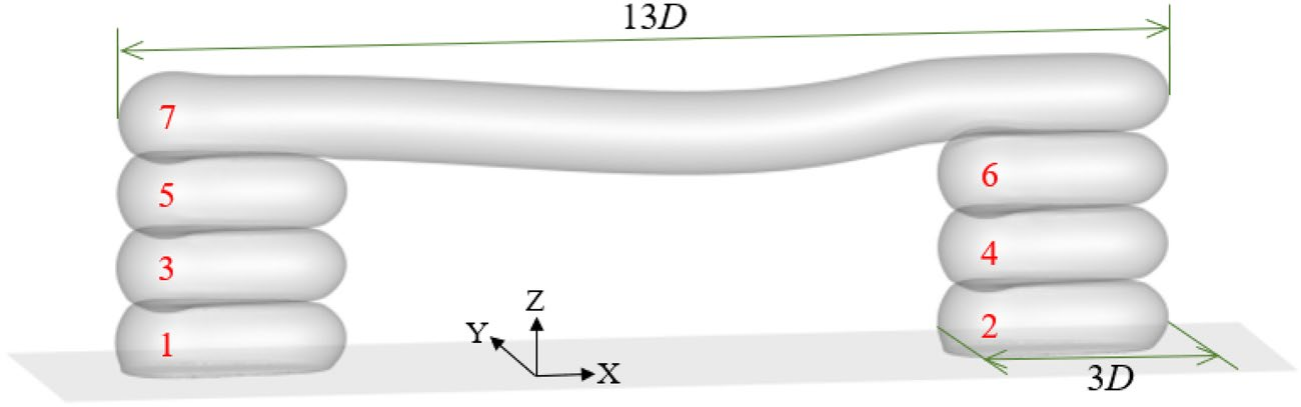
A schematic of the horizontal extension printing.

Figure 7 shows a schematic of the horizontal extension printing. The part was divided into four layers, and the bottom three layers were piers. The width of the pier and bridge deck were 3*D* and 13*D*, respectively. Furthermore, when printing each layer, the height of the volume source from the substrate was 0.4*D*, 1.3*D*, 2.2*D*, and 3.1*D*. The red numbers in the figure represent the printing order of each component in the part, and the position of the numbers was also the starting point of the volume source movement. The part was positioned at the center of the computational domain, allowing an additional 3 s for cooling the printed part. The velocity *U*_s_ of the volume source and the temperature *T*_*inj*_ of the generated material are 0.2 m/s and 215 °C respectively.

Polylactic acid filament was widely utilized in the FFF process, serving as the forming material, and suggesting that the ambient gas was air. The main physical parameters are shown in Table 2. Subscript *a* and *p* represent ambient gases and materials, respectively.

**Table 2:** The main physical parameters.

Parameter	Notation	Value	Unit
Air density	*ρ*_*a*_	0.9	kg/m^3^
Air viscosity	*µ*_*a*_	2.3 × 10^–5^	Pa s
Air thermal conductivity	*λ*_*a*_	0.034	W/(m K)
Air specific heat capacity	*c*_*p,a*_	1000	J/(kg K)
Material density	*ρ*_*p*_	1240	kg/m^3^
Material viscosity	*µ*_*p*_	20–5000	Pa s
Material thermal conductivity	*λ*_*p*_	0.195	W/(m K)
Material specific heat capacity	*c*_*p,p*_	2000	J/(kg K)
Surface tension coefficient	*σ*	0.04	kg/s^2^

### Process evolution

**Figure 8:**
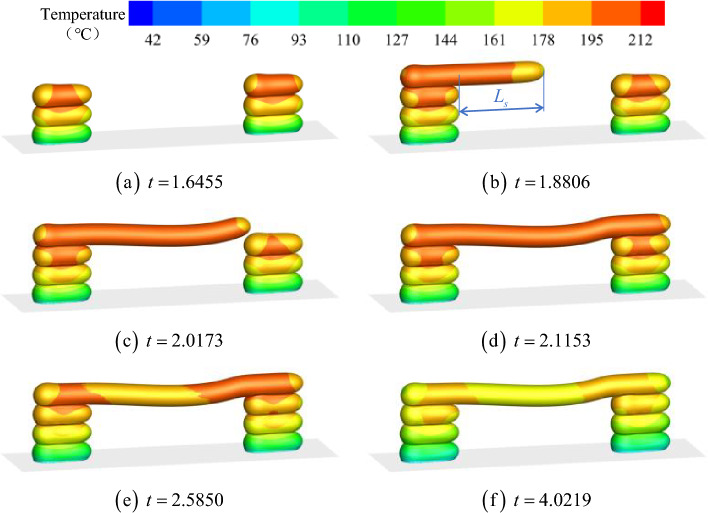
Evolution of material deposition morphology and surface temperature.

Figure 8 shows the evolution of material extension morphology and surface temperature. After the pier on both sides was printed (*t* = 1.6455), the overall temperature of each layer gradually decreased from top to bottom, and the temperature at the top was higher compared with the bottom side. At *t* = 1.8806, it is evident that the bridge deck was relatively straight. However, when the process advanced to *t* = 2.0173, the bridge deck experienced sagging deformation. Until *t* = 2.1153, one layer of material on the bridge deck was formed. Thus, during the subsequent cooling process, the temperature of the bridge deck continuously decreased. Figure 8e and f indicated that the temperature near the middle of the bridge deck decreased faster than on both sides, and heat appeared to spread toward the piers on both sides. Compared with Fig. 8d and f, there were insignificant changes in the morphology of the bridge deck during cooling.

**Figure 9:**
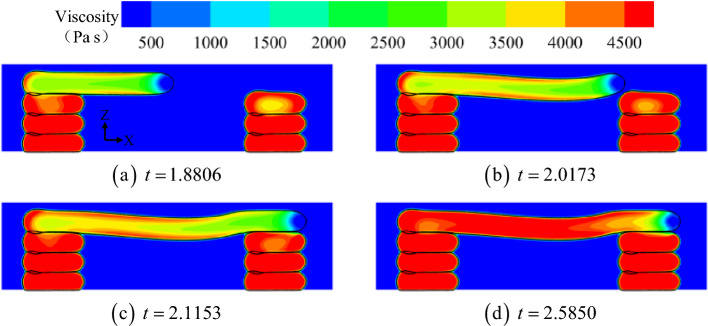
Viscosity contours in the Y-axis midpoint plane.

To explore the underlying physical mechanism of material behavior in Figs. 8 and 9 exhibited the viscosity contours in the Y-axis midpoint plane. The four moments in Fig. 9 corresponded to Fig. 8b–e, respectively. The viscosity on the surface of the overhanging portion of the bridge deck rapidly increased during printing. However, because the volume source moved faster than the heat dissipation rate inside the material, given the large range of the internal area, the viscosity remained at ~ 3000 Pa s, indicating a molten or semi-molten state (at *t* = 1.8806 and *t* = 2.0173). Additionally, the deflection of the overhanging portion of the bridge deck increased with increasing horizontal extension distance. When the internal viscous force and the solid stress on the material surface are no longer balanced with gravity, sagging deformation occurs (*t* = 2.0173). Figure 9c and d corresponded to the cooling and solidification of the material. Figure 9c depicts that the temperature near the middle of the bridge deck dropped faster than at the pier.

**Figure 10:**
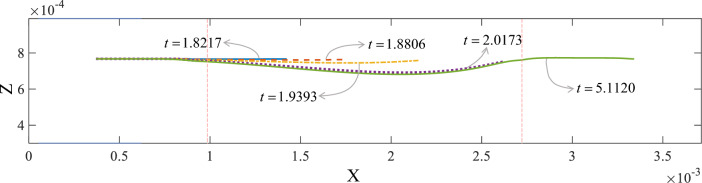
Evolution of the axis morphology of the bridge deck.

The axis morphology of the bridge deck at different times during printing is depicted in Fig. 10. The solid blue line illustrates that although the sustained distance is very short, the molten material remained horizontally extended over time. Afterward, the overhanging portion of the bridge deck gradually sagged in the Z-axis direction. The sagging bridge deck formed an approximately smooth deflection curve.

**Figure 11:**
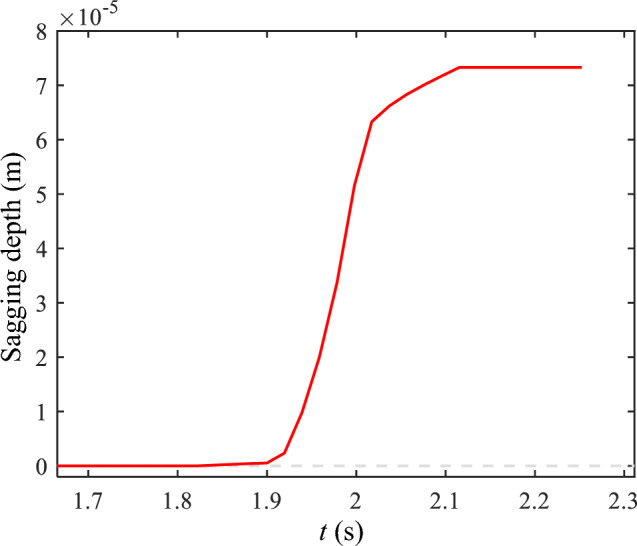
The sagging depth of the bridge deck.

To quantitatively evaluate the process of bridge deck deflection, the variation of bridge deck sagging depth over time is plotted in Fig. 11. Before *t* = 1.8217, the sagging depth was zero. Initially, the bridge deck sagged slowly at *t* = 1.8999, then accelerated significantly (*t* = 2.0173), tending slowly downward at *t* = 2.1153. Finally, after one layer of the material on the bridge deck was printed, the sagging depth remained constant.

### The effect of printing temperature

In self-supporting printing, two decisive factors were the thermodynamic and kinetic behavior of the material. Different printing temperatures and velocities led to distinct thermal and flow fields in the domain, consequently affecting the behavior of the material. Section "The effect of printing temperature" and "The effect of printing velocity" therefore examined the influence mechanism of printing temperature and printing velocity, respectively, on the horizontal extension process of the molten material.

**Figure 12:**
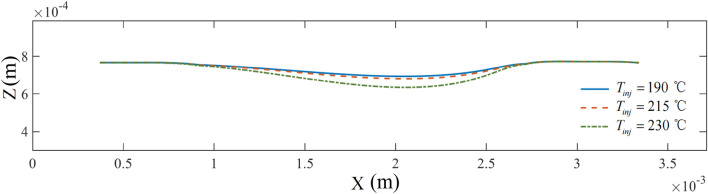
The axis morphology of the bridge deck at different printing temperatures.

Figure 12 compares the axis morphology of the bridge deck under different printing temperatures. Here, we maintained a printing velocity of 0.2 m/s. As the printing temperature increased, the deflection of the overhanging portion increased, and the molten material was horizontally extended. Furthermore, when the printing temperature increased from 190 to 215 °C, the increase in deflection was relatively small. However, when the printing temperature was further increased to 230 °C, the change in deflection was more significant. Unlike the overhanging portion, each printing temperature curve essentially coincided on both sides of the piers.

**Figure 13:**
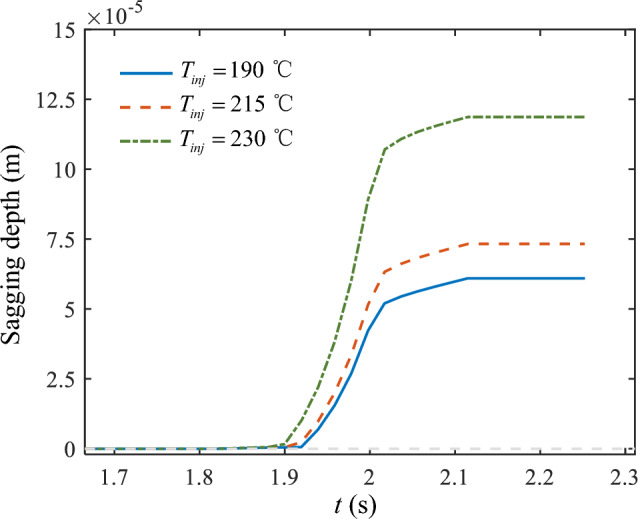
The sagging depth of the bridge deck at different printing temperatures.

Figure 13 shows the sagging depth of the material in the forming process at different printing temperatures. Additionally, regardless of the printing temperature, the sagging depth of the material underwent a gradual development process, ultimately becoming a constant value. Consistent with the changing trend of the axis morphology of the molten material in Fig. 12, the depth of material sagging increased simultaneously with increasing printing temperature. Thus, the rate of increase was intensified.

**Figure 14:**
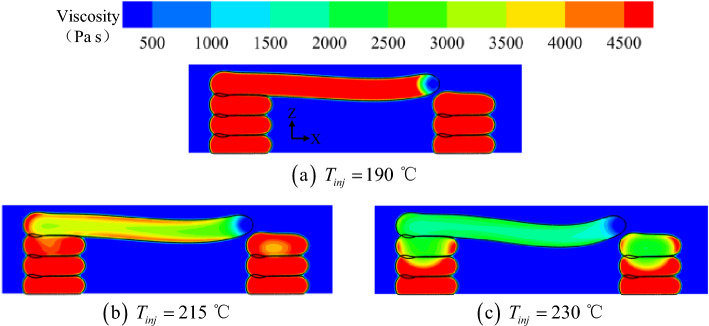
Viscosity contours in the Y-axis midpoint plane at different printing temperatures.

The viscosity contours in the Y-axis midpoint plane at different printing temperatures are shown in Fig. 14. Furthermore, when printing at the same position, the viscosity of the bridge deck material varied significantly at different printing temperatures. For lower printing temperatures (190 °C), the semi-molten material solidified quickly after generation. At this point, due to the strong intermolecular bonding force, the sagging deformation of the material during horizontal extension was relatively minimal. Subsequently, when the printing temperature was very high (230 °C), the temperature of the molten material did not decrease promptly during the brief deposition and spreading period. It was observed that, unlike Fig. 14b, the viscosity in Fig. 14c was still very low even on the surface of the overhanging portion of the material. Thus, due to the absence of solid stress on the surface and the low internal viscosity, the sagging deformation of the material during horizontal extension was the most significant.

**Figure 15:**
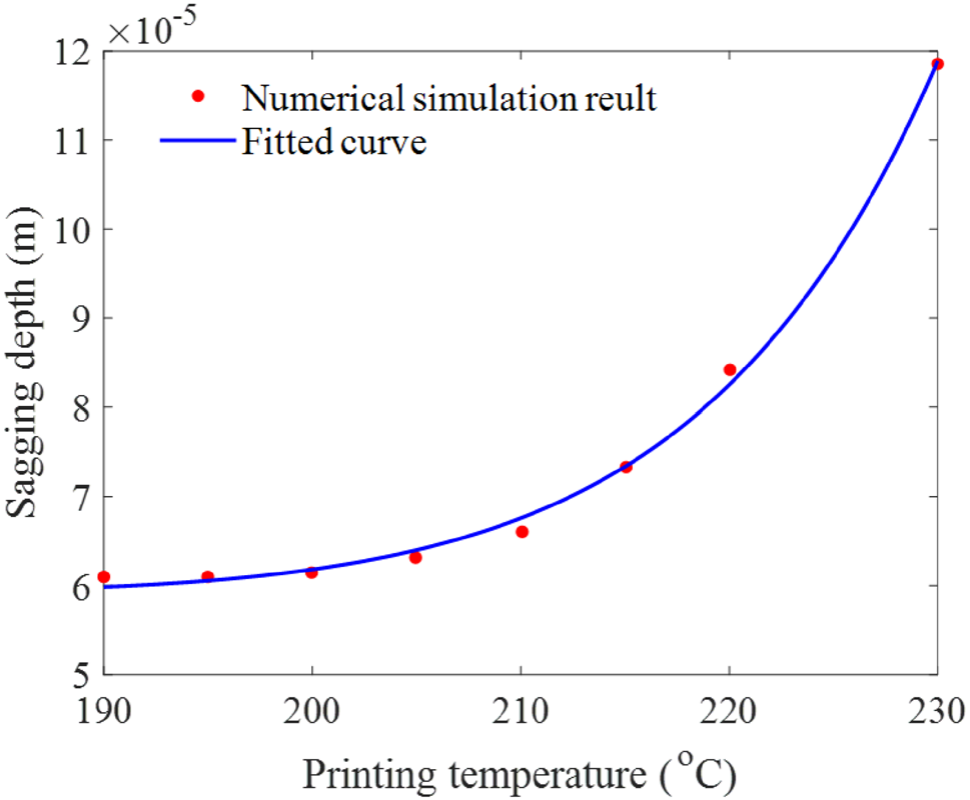
The maximum sagging depth of the bridge deck at different printing temperatures.

Figure 15 summarizes the variation of the maximum sagging depth of the bridge deck with the printing temperature. Consistent with the previous findings, the depth of material sagging increased monotonically in the range of 190 °C < *T*_*inj*_ < 230 °C. The variation trend of sagging depth *d* with printing temperature *T*_*inj*_ in Fig. 15 approximately satisfied a modified exponential equation (i.e., the solid blue line):

11 $$ d = \left[ {0.102\left( {T_{inj} - 190} \right)e^{{0.067\left( {T_{inj} - 190} \right)}} + {59}{\text{.825}}} \right] \times 10^{ - 6} \quad \left( {190 < T_{inj} < 230} \right) $$

This equation indicates that as the printing temperature increases from 190 to 230 °C, the sagging depth of the material increases slowly and then rapidly. The fitted curve suggests an inflection point for the printing temperature, around 210 °C. When the printing temperature is lower than this point, the sagging depth is insensitive to the printing temperature, while it is quite sensitive when the printing temperature is higher. This results from the balance between the viscous force and gravity force. When the printing temperature is high, the viscosity becomes relatively low, and thus the gravity force is dominated, resulting in increased sagging displacement. Additionally, the extruded material at a higher temperature needs more time to cool down, which further adds more displacement to the sagging deformation. Consequently, the sagging depth increases more and more rapidly as the printing temperature increases.

### The effect of printing velocity

**Figure 16:**
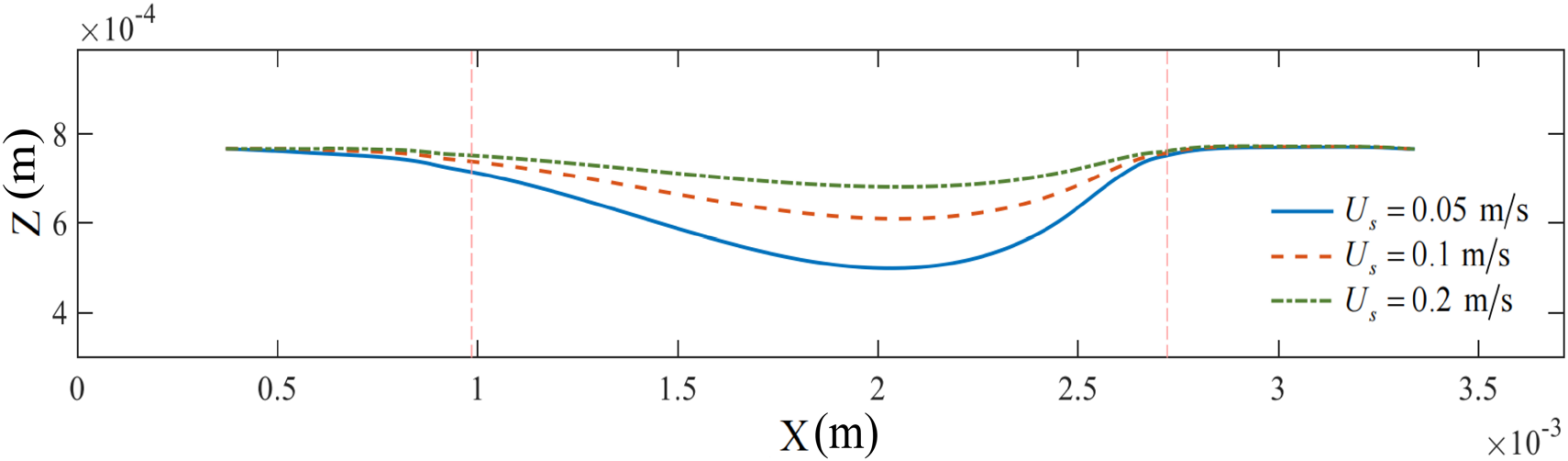
The axis morphology of the bridge deck at different printing velocities.

In self-supporting printing, printing velocity is also a critical process parameter. To examine the effect of printing velocity, we varied the velocity from 0.05 to 0.2 m/s while maintaining the printing temperature at 215 °C. Figure 16 suggests that the lower the printing velocity, the greater the deflection of the overhanging portion of the bridge deck. Unlike the situation where the three curves at the right pier essentially coincided, the curvature of the left end of the curve increased with the decreasing printing velocity. This indicated that at a lower printing velocity (*U*_s_ = 0.05 m/s), the bending degree of the bridge deck was the highest at the left pier and the overhanging portion. Representing *U*_s_ = 0.05 m/s, the large curvature of the blue solid line on the left side was due to the vertical downward drag of the overhanging portion. Conversely, it was attributable to the slower printing velocity, enhancing the interlayer molten material to exhibit a relatively sufficient interaction time, resulting in significant deformation of the underlying material.

**Figure 17:**
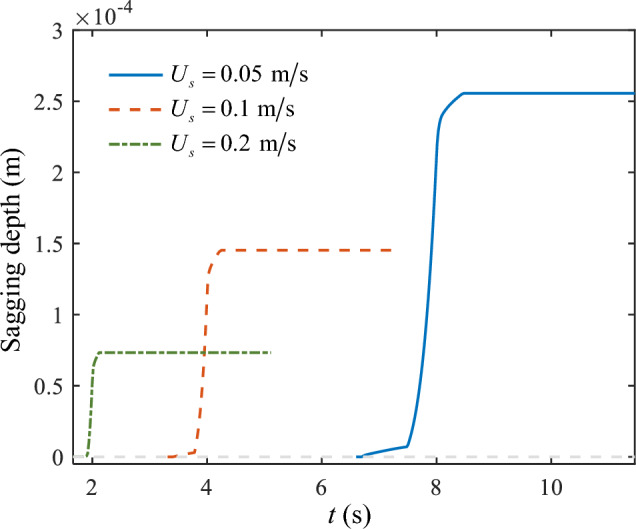
The sagging depth of the bridge deck at different printing velocities.

The variation process of the bridge deck sagging depth with time is plotted in Fig. 17. Furthermore, due to different printing velocities, the time required to print to the same location varied. This resulted in less overlap in the evolution time domain of the three curves (Fig. 17). However, corresponding to different printing velocities, the basic trend of the three curves was similar. Among them, the smaller the printing velocity, the greater the final depth of the bridge deck.

**Figure 18:**
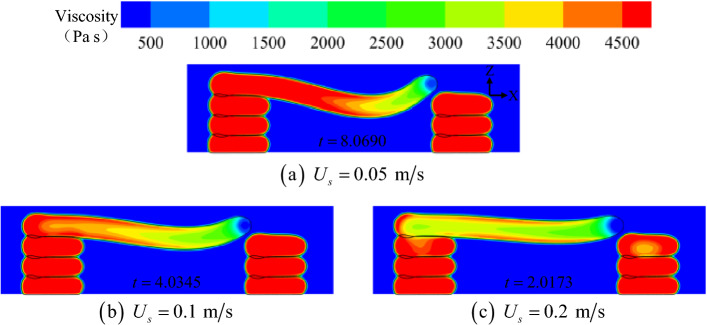
Viscosity contours in the Y-axis midpoint plane at different printing velocities.

Figure 18 shows the viscosity contours in the Y-axis midpoint plane. Notably, when printing to approximately the same location, a smaller printing velocity resulted in a larger high-viscosity area inside the bridge deck. This occurred because the shear rate of the material was lower at a slower printing velocity, allowing for more adequate cooling time. However, at higher printing velocities (*U*_s_ = 0.2 m/s), a significant portion of the material remained in a molten or semi-molten state.

**Figure 19:**
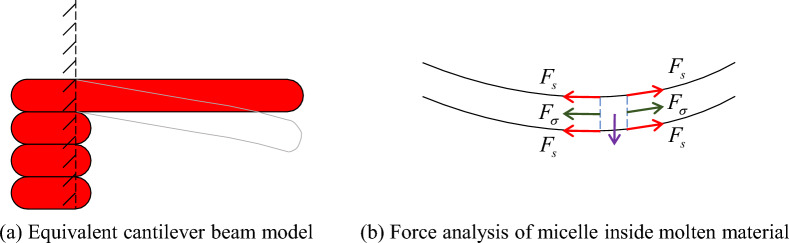
Analysis of the force mechanism of the bridge deck.

For a slow printing velocity, an equivalent cantilever beam structure was formed between the bridge deck and the left pier after solidification (Fig. 19a), and the free end of the bridge deck exhibited a significant trend of bending. As the printing velocity increased, the tensile force (normal stress) between the micelles inside the molten material increased under strong inertia. Subsequently, combined with the solid stress of the thin layer on the surface of the material, the bridge deck exhibited a specific self-supporting potential (Fig. 19b). The printing velocity fundamentally affected the relaxation time of the thermal and dynamic properties of the material.

**Figure 20:**
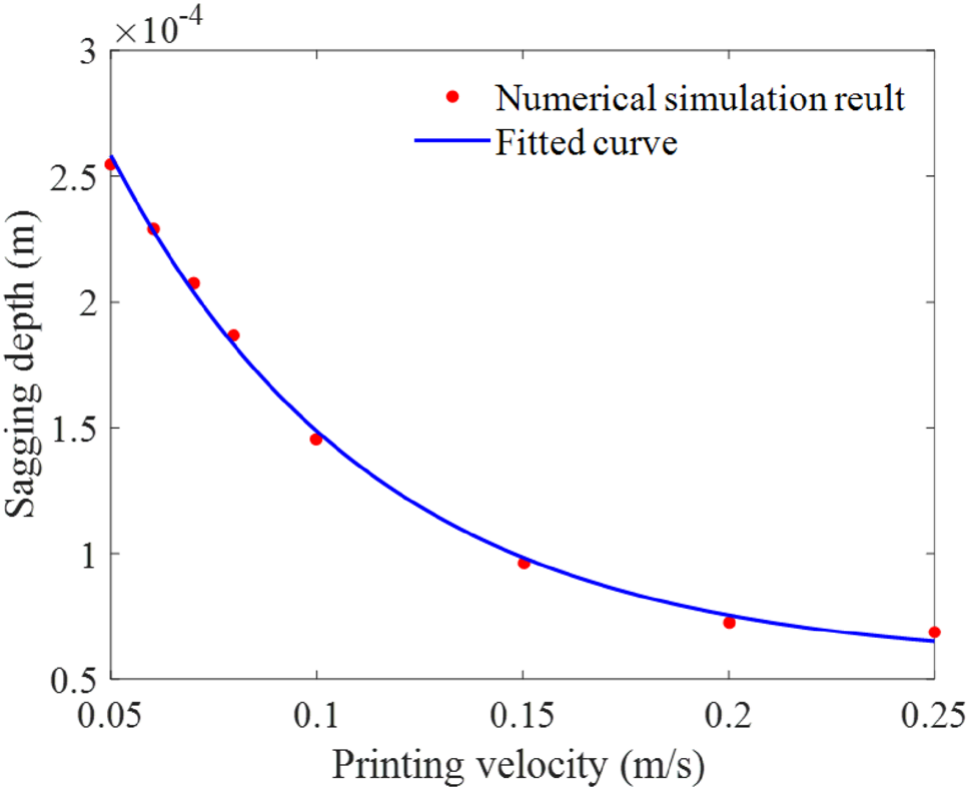
The maximum sagging depth of the bridge deck at different printing velocities.

The variation trend of the maximum sagging depth of the bridge deck with the printing velocity is plotted in Fig. 20. The velocity ranged from 0.05 to 0.25 m/s. Notably, when the printing velocity increased, the sagging depth of the material continuously decreased, with a decreasing reduction rate. The variation trend was fitted using another modified exponential equation, represented by the blue solid line in the figure:

12 $$ d = \left( {441.655e^{{ - 15.620U_{s} }} + 56.086} \right) \times 10^{ - 6} \quad \left( {0.05 < U_{s} < 0.25} \right) $$

This equation indicates that the sagging depth decreases rapidly and then slowly as the printing velocity increases from 0.05 to 0.25 m/s. When the printing velocity is lower, the hot nozzle maintains the extruded material at a high-temperature level for more time, as a consequence, the sagging displacement of the extruded material is larger. The aforementioned maintaining time decreases in an approximately inversely proportional relationship with the printing velocity, and the displacement presents an approximately squared relationship with the time according to Newton's second law. Therefore, the sagging depth decreases slower and slower as the printing velocity increases.

### Verification

**Figure 21:**
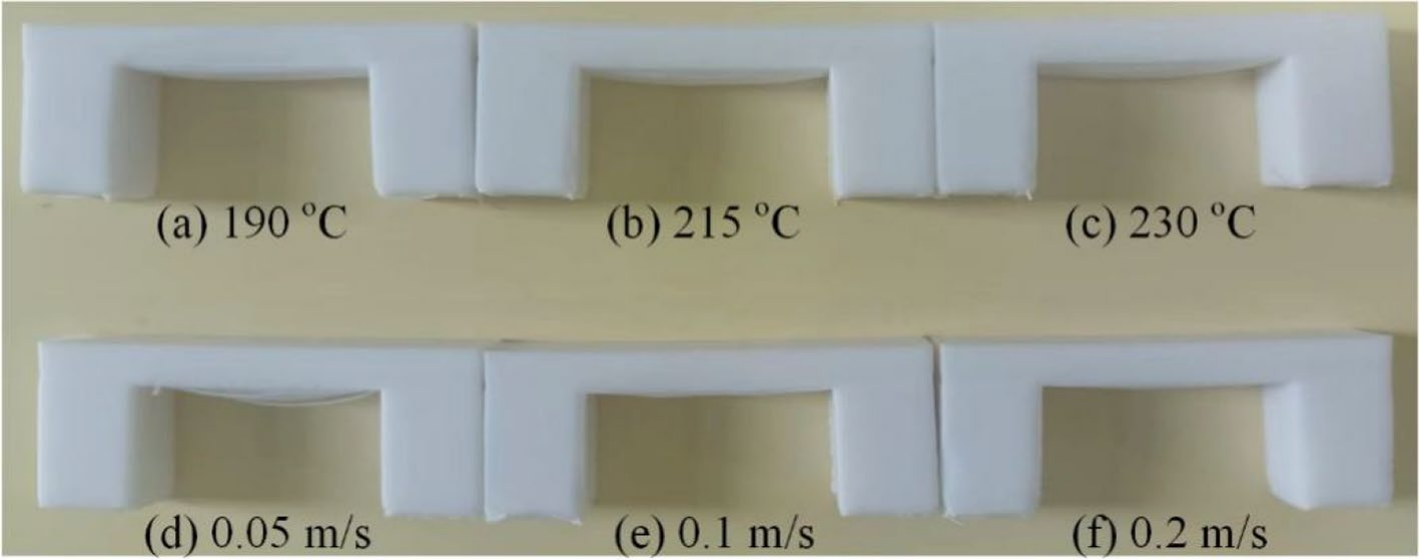
Sagging depth of the bridge deck at different (**a**–**c**) printing temperatures and (**d**–**f**) printing velocities.

Due to the limitations of the presented numerical method and the printer, the huge computational cost limits us to simulate cases with a large size, and the printer cannot properly print a part with a millimeter-level size. Here, we carried out a compromised comparison, where the computational configurations remain as they were presented aforementioned, and the experiments were conducted at a larger scale of 10 × 20 × 45 mm as presented in Fig. 21. Due to the difference in the scale, the goal of this comparison is to qualitatively and primarily validate the numerical method. The predicted and experimental results are listed in Table 3, where the sagging depths of the numerical and experimental cases are obtained at the deck length *L* of 1.75 mm and 25 mm, respectively. The results indicate that the presented numerical method can give similar tendencies of the sagging depth changing with the printing temperature and velocity as those given by the experiments. Furthermore, we compared the sagging ratio that excludes the influence of the deck length. The simulated sagging ratio is 3.42–6.82% as the printing temperature rises from 190 °C ~ 230 °C and 14.76% ~ 4.31% as the printing velocity increases from 0.05 m/s ~ 0.2 m/s. The experimental sagging ratio is 3.84% ~ 6.32% and 13.48% ~ 4.68% as the printing temperature and velocity vary in the corresponding ranges.

**Table 3:** Sagging depth comparison between the simulations and the experiments.

	Printing temperatures (°C)			Printing velocities (m/s)		
	190	215	230	0.05	0.1	0.2
Simulation@*L* = 1.75 mm *d*(μm)	60	73	119	258	149	76
Experiment@*L* = 25 mm *d*(μm)	960	1140	1580	3370	2190	1170
Simulated sagging ratio *d*/*L*	0.0342	0.0420	0.0682	0.1476	0.0850	0.0431
Experimental sagging ratio *d*/*L*	0.0384	0.0456	0.0632	0.1348	0.0876	0.0468

## Conclusion

A comprehensive model was developed for the FFF 3D printing process by applying the front-tracking method for multiphase coupling, and a volume source for the nozzle. The common physical behaviors of polymer materials used in the FFF process induced by heat and mass transfer, moving interface, rheology, surface tension, and gravity were successfully simulated. Upon the model, fully resolved simulations were conducted for the horizontal extension structure.

Overhang structures were prevalent in the FFF process, and the simulation of the horizontal extension printing indicated that the molten material exhibited a specific self-supporting forming ability. However, for different process parameters, the ultimate distance of self-supporting printing varied. Furthermore, during the self-supporting printing process of large-span horizontally extended structures, the bridge deck underwent a gradual evolution from initial straight extension to sagging deformation, ultimately presenting a curved shape. The distance of the straight extension was inversely proportional to the depth of the sagging deformation. The printing temperature affected the curing time of the material, and the intermolecular force of the materials in different phase states determined the final shape of the bridge deck. In the temperature range of 190 °C–230 °C, the sagging deformation depth of the material increased slowly with increasing printing temperature and then accelerated. Fundamentally, the printing velocity affected the relaxation time of the material thermal and dynamic properties. Under different printing temperatures, the stress mechanism of the bridge deck varied. In the velocity range of 0.05–0.25 m/s, the sagging deformation depth of the material consistently decreased with increasing printing velocity, with a decreasing rate.

## Data Availability

Data will be available on request.
